# Effectiveness and safety of proton therapy in intracranial meningioma treatment: a systematic review and meta-analysis

**DOI:** 10.1007/s10143-026-04319-5

**Published:** 2026-05-09

**Authors:** Jeremiah Hillkiah Wijaya, Abdelrahman Ramadan Elashry, Sarmad Javaid, Mohga Yasser, Bismon Jibu, Bharath Narayanan, Daniela A. Perez-Chadid, Aafreen Azmi, Juan Pablo Avila-Madrigal, Elizabeth E. Ginalis, Anil Nanda

**Affiliations:** 1 School of Public Health and Preventive Medicine, Monash University, Melbourne, VIC Australia; 2 College of Medicine, University of Sharjah, Sharjah, UAE; 3 Sharif Medical and Dental College, Lahore, Pakistan; 4 Far Eastern Medical College, Khabarovsk, Russia; 5 Department of Neurosurgery, Rutgers New Jersey Medical School, Rutgers University, Newark, NJ USA; 6 Department of Neurosurgery, Robert Wood Johnson Medical School, Robert Wood Johnson University Hospital, Rutgers University, New Brunswick, NJ USA; 7 Anatomy Department, Universidad de los Andes School of Medicine, Bogota, Colombia

**Keywords:** Intracranial meningioma, Proton therapy, Radiotherapy, Stereotactic radiosurgery

## Abstract

Meningiomas are the most common primary intracranial tumors, often treated surgically. However, complete resection is frequently limited by proximity to critical structures, necessitating adjuvant or definitive radiotherapy. Proton therapy offers dosimetric advantages over photon-based radiotherapy, particularly in sparing adjacent normal tissues. This study aims to systematically evaluate the effectiveness and safety of proton therapy for intracranial meningiomas across tumor grades and clinical scenarios. A systematic review and meta-analysis was conducted according to PRISMA 2020 guidelines using PubMed, EMBASE, Scopus, Web of Science, and Cochrane from inception to November 10, 2025. Studies were eligible if they reported clinical outcomes of proton therapy in ≥ 10 adult meningioma patients. Data extraction and risk-of-bias assessment were performed independently by two reviewers. Pooled complication rates and survival outcomes were calculated using random-effects models. Nineteen studies involving 1,431 patients were included. WHO Grade I tumors comprised 70.6% of cases; Grades II/III made up 25.2% and 4.2%, respectively. The most common proton dose regimens ranged from 13 to 70.2 Gy (RBE). The pooled complication rate was 16% (95% CI 5–27; *p* < 0.001; I² = 98.5%). Nine studies reported a statistically significant 5-year overall-survival proportion of 91% (95% CI 88–94; *p* < 0.001; I² = 49.3%). Radiologic local control averaged 71% (95% CI 50–86; I² = 88.2%). Proton therapy provides effective tumor control with acceptable toxicity, especially for low-grade or anatomically complex meningiomas. It is a valuable option for select patients, though further prospective studies are needed to optimize dosing and assess long-term outcomes.

## Introduction

Meningiomas are the most common primary intracranial tumors, accounting for nearly 39% of all central nervous system neoplasms, with an incidence of 8.6 per 100,000 person-years [1]. While most meningiomas are histologically benign (WHO Grade I), up to 20–30% are atypical or anaplastic (Grade II/III), associated with higher recurrence rates and poorer prognosis [1]. Management options include observation, radiotherapy, and surgical resection; for surgically accessible and symptomatic lesions, gross total resection remains the mainstay of treatment [2]. However, complete resection is often limited by tumor proximity to critical neurovascular structures such as the optic chiasm, cranial nerves, and brainstem, making adjuvant or definitive radiotherapy essential, particularly after subtotal resection or in higher-grade tumors [2].

Among radiotherapy modalities, intensity-modulated radiotherapy (IMRT) and stereotactic radiosurgery (SRS) are widely utilized due to their precision and favorable safety profiles [3, 4]. Proton therapy, however, has gained increasing attention for its unique Bragg peak property, allowing high-dose deposition within the tumor while minimizing radiation to surrounding healthy brain tissue. This is particularly advantageous for tumors located in complex skull base regions, pediatric or young adult patients with long expected survival, or in cases requiring re-irradiation. Although proton therapy is dosimetrically superior to photon-based techniques like IMRT, whether this translates into improved clinical outcomes or reduced toxicity in meningioma patients remains a topic of ongoing investigation [4]. Moreover, access to proton therapy remains limited due to the high cost and geographic concentration of facilities, making it essential to assess whether the benefits justify the resource investment.

While previous studies have reported encouraging outcomes with proton therapy in intracranial meningiomas, findings have often been based on small cohorts or single-center experiences. No previous meta-analysis has comprehensively quantified both toxicity and survival outcomes of proton therapy across meningioma grades and clinical scenarios. A comprehensive synthesis of current evidence is lacking, particularly with respect to treatment efficacy, overall survival, and toxicity profiles across different clinical scenarios and tumor grades. Therefore, this systematic review and meta-analysis aim to evaluate the effectiveness and safety of proton therapy in the treatment of intracranial meningiomas.

## Method

### Literature search

This systematic review and meta-analysis was conducted in accordance with the 2020 PRISMA guidelines [5]. A thorough literature review was conducted using five electronic databases: PubMed, EMBASE, Scopus, Web of Science, and Cochrane which included publications from inception to November 10, 2025. The literature search utilized both keywords and controlled vocabulary for “proton therapy,” “intracranial meningioma,” and “radiation therapy.” Detailed search terms are presented in Table 1.

**Table 1:** Search strategy and used database

Online Registry	Search Strategy
PubMed	(“Meningioma“[MeSH Terms] OR “meningioma*“[Title/Abstract] OR “intracranial meningioma“[Title/Abstract] OR “brain meningioma“[Title/Abstract]) AND (“Proton Therapy“[MeSH Terms] OR “Proton Therapy“[Title/Abstract] OR “proton-beam therapy“[Title/Abstract] OR “proton-beam therapy“[Title/Abstract] OR “proton radiotherapy“[Title/Abstract] OR “proton irradiation“[Title/Abstract] OR “proton beam radiation“[Title/Abstract] OR “pbt“[Title/Abstract]) AND (“effectiveness“[Title/Abstract] OR “efficac*“[Title/Abstract] OR “outcome*“[Title/Abstract] OR “survival“[Title/Abstract] OR “safety“[Title/Abstract] OR “toxicit*“[Title/Abstract] OR “adverse“[Title/Abstract] OR “complication*“[Title/Abstract])
EMBASE	((‘proton therapy’/exp OR ‘proton beam radiotherapy’/exp OR (proton NEAR/3 (therapy OR radiotherapy OR irradiation)): ti, ab OR ‘proton beam’:ti, ab) AND (‘meningioma’/exp OR meningioma*:ti, ab OR ‘meningeal tumor*’:ti, ab) AND (intracranial: ti, ab OR brain*:ti, ab) AND (‘efficacy’/exp OR ‘safety’/exp OR efficacy: ti, ab OR effectiveness: ti, ab OR safety: ti, ab OR toxic*:ti, ab OR outcome*:ti, ab OR adverse: ti, ab)) AND [humans]/lim AND [english]/lim
Scopus	TITLE-ABS-KEY (meningioma* OR “intracranial meningioma” OR “brain meningioma”) AND TITLE-ABS-KEY (“proton therapy” OR “proton beam therapy” OR “proton radiotherapy” OR “proton irradiation” OR “proton beam radiation” OR pbt) AND TITLE-ABS-KEY (effectiveness OR efficacy OR outcome* OR survival OR safety OR toxicity OR adverse OR complication*)
Web of Science	proton therapy OR radiotherapy OR irradiation OR beam therapy AND intracranial OR cranial OR skull base OR brain AND meningioma AND outcome* OR survival OR toxicity OR adverse event* OR complication* AND meninigioma
Cochrane	meningioma* OR “intracranial meningioma” OR “brain meningioma” AND “proton therapy” OR “proton beam therapy” OR “proton radiotherapy” OR “proton irradiation” OR “proton beam radiation”

### Eligibility criteria

All studies that documented outcomes after proton therapy for adult meningioma patients were considered. Studies that reported combined proton–photon cohorts without separate outcome data for the proton subgroup were excluded from the quantitative synthesis and are listed among excluded articles. Studies that provided extractable proton-only subgroups were included using only the proton-specific data. Study eligibility included non-randomized interventional studies, cohort studies, and case series with a minimum of 10 patients. Only studies published in English were included. The exclusion criteria consisted of review articles, editorials, case studies, conference publications without full-text access, and publications that overlapped.

### Study selection & data extraction

The four reviewers (JH, SJ, MK, and AR) independently screened and selected studies by reviewing titles and abstracts and then the full texts of eligible studies. Differences in study selection were resolved with group discussion. Two reviewers (JH and SJ) independently and in a blinded manner with respect to each other’s ratings completed a standardized data-extraction form that includes study design, patient age and sex, tumor type, specifics of proton therapy, follow-up length, and outcomes. For safety, the primary outcome of interest was the complication rate, which in this case meant adverse events associated with the treatment. Regarding efficacy, the included outcomes were the overall survival after five years and the locally controlled rate determined by serial follow-up radiological imaging.

### Risk of bias assessment

Each study included in the analysis was evaluated for bias using the Risk Of Bias In Non-randomized Studies of Interventions (ROBINS-I) tool. [6] The tool assesses bias in confounding, selection, classification of the intervention, deviations from the intended intervention, missing data, outcome measurement, and reporting. Five reviewers (SJ, MK, AR, BJ, and JH) independently and initially blinded to each other’s judgments evaluated risk of bias; disagreements were resolved through discussion and, when necessary, consultation with a senior reviewer.

### Statistical analysis

Effect measures were calculated using a meta-analysis of proportions, conducted in RStudio (version 2024.03.1 + 402) with the meta v7.0-0, metafor v3.8-1, dmetar v0.1.2. All analyses were performed using a random-effects model regardless of the degree of heterogeneity to account for potential variability across studies, implementing an inverse-variance random-effects approach with the DerSimonian–Laird estimator for between-study variance; sensitivity analyses using the Hartung–Knapp–Sidik–Jonkman adjustment produced similar results. Pooled estimates were reported with 95% confidence intervals (CIs), and statistical heterogeneity was assessed using the I² statistic and Cochran’s Q test. To assess the robustness of the pooled estimates and identify potential sources of significant heterogeneity, a leave-one-out sensitivity analysis was conducted. This strategy involved iteratively recalculating the meta-analysis while omitting one study at a time to determine the specific influence of individual studies on the overall effect size and heterogeneity metrics. A p-value of less than 0.05 was considered statistically significant. Publication bias was evaluated through visual inspection of funnel plots as well as formal tests, including Egger’s regression asymmetry test and Begg’s rank correlation test.

## Results

### Study selection

**Fig. 1:**
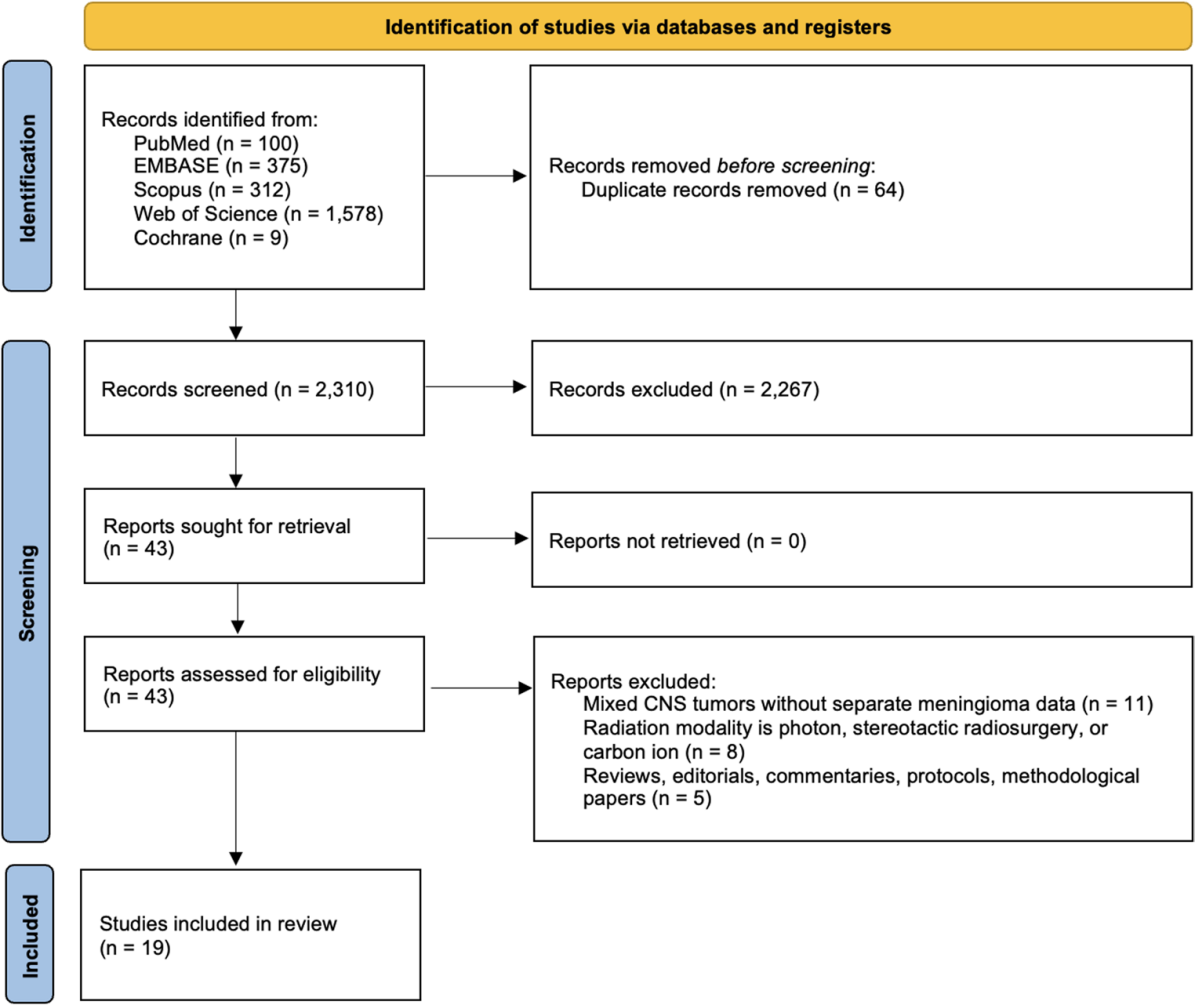
PRISMA flow diagram of study selection for proton therapy in intracranial meningioma

As illustrated in Fig. 1, we identified 2,374 records across five databases, including PubMed (*n* = 100), EMBASE (*n* = 375), Scopus (*n* = 312), Web of Science (*N* = 1,578), and Cochrane (*n* = 9). We removed 64 duplicates, leaving 2,310 unique records for title/abstract screening. Of these, 2,267 were excluded, and 43 full-text articles were retrieved and assessed for eligibility (none were unavailable). Seventeen reports were then excluded for the following reasons: mixed CNS tumor cohorts without separate meningioma data (*n* = 11), use of non-proton radiation modalities (photon, stereotactic radiosurgery, or carbon ion; *n* = 8), and non-original research formats (reviews, editorials, protocols; *n* = 5). This process yielded 19 studies for inclusion in the systematic review and meta-analysis [7–25].

### Patient and tumor characteristics

The 19 studies collectively involved 1431 patients with intracranial meningiomas treated with proton therapy (Table 2). The sample was made up of 53.7% primary disease cases and 46.3% recurrent/progressive disease cases, with only 3 patients receiving re-irradiation. Tumor grading distribution showed that WHO Grade I meningiomas were most commonly represented (70.6%), followed by Grade II (25.2%) and Grade III (4.2%). The general protocol for dose regimen described was 1.8– to 2.0-Gy fractions 5 days per week to a total dose of 50–60 Gy. The dose regimen for these studies ranged from 50.4 to 66.6 Gy for Grade I, while 54–70.2 Gy for Grade II/III.

**Table 2:** Baseline demographics, treatment parameters, and key toxicities in proton-therapy cohorts for intracranial meningioma

Study ID	*n*	Primary	Recurrent	WHO I/II/III	Dose (Gy RBE/CGE)	FU (mo)	Key ≥ G3 toxicity (*n*)
Vernimmen 2001	27	27	0	NS	54–61.6 CGE (16–27 fx)	12	Transient CN neuropathy (2), hearing loss (1), epilepsy (1)
Weber 2012	39	7	32	23/9/2	56 Gy (1.8–2 Gy RBE/fx)	62	Optic neuropathy (3), brain necrosis (2)
Weber 2004	16	11	5	11/2/–	56 CGE (1.8–2 CGE/fx)	34	Visual loss (1), hemiparesis (1)
Murray 2017	96	53	43	61/33/2	54 Gy (G I) – 62 Gy (G II/III)	12	Optic toxicity (14), pituitary (10)
Shafie 2018	110	42	68	60/7/1	54 Gy proton; 18 Gy carbon	47	Radionecrosis (3)
Slater 2012	72	22	50	NS	50.4–70.2 Gy	74	Optic symptoms (3), edema (2)
Vlachogiannis 2017	170	44	126	NS	21.9 Gy (5–6 Gy/fx)	84	Radiation necrosis (5)
Halasz 2011	50	32	18	NS	13 Gy (10–15.5)	32	Cranial neuropathy (29)
Gudjonsson 1999	19	19	0	NS	24 Gy (4 × 6 Gy)	36	–
Boskos 2009	24	24	0	NS	34 CGE (mean)	32	Radiation necrosis (1)
McDonald 2015	22	12	10	NS	63 Gy	39	Radiation necrosis (1)
Hug 2000	31	16	15	NS	40–72 Gy (mean 58 CGE)	59	Radiation necrosis (2)
Iannalfi 2024	167	NR	NR	NS	55.8 Gy (benign); 66 Gy (atypical)	41	Grade-3 acute (2), late (3)
Krcek 2023	200	111	89	140/55/5	54 Gy (WHO I); 60 Gy (WHO II/III)	65	≥ G3 late events (24)
Song 2021	77	77	0	NS	NR	26	≥ G2 toxicity (8)
Holtzman 2022	59	59	0	59/–/–	50.4 Gy (48.6–61.2)	75	≥ G3 toxicity (1)
Sato 2021	27	27	0	27/–/–	52 Gy (45–66)	126 (max 301)	Radiation necrosis (1)
Champeaux-Depond 2021	193	0	193	171/13/9	54 Gy (54–59.4)	53	Treatment-related toxicity (23)
Scartoni 2021	32	0	32	10/22/–	54 Gy (50.4–60)	27	Grade ≥ 3 toxicity (2)

*FU* follow-up, *NS* not specified, *NR* not reported, *CGE/RBE* cobalt-gray equivalent/relative biologic effectiveness

### Adverse events and complication profile

Adverse effects observed included acute, treatment-period toxicities in 41.0% of patients (alopecia, *n* = 158; skin erythema/irritation, *n* = 115; fatigue, *n* = 66) and late-onset toxicities in 11.9% of patients (optic neuropathy, *n* = 17; pituitary dysfunction, *n* = 14; radiation necrosis, *n* = 15) [7–25]. Five-year overall survival after proton therapy was 91% [95% CI 88–94] for WHO Grade I and 70–85% for WHO II/III. When toxicity grades were reported, most adverse events were low grade (CTCAE grade 1–2), such as alopecia, skin erythema/irritation, fatigue, headache, and nausea. Across all 19 studies (*n* = 1431), at least 36 high-grade (grade ≥ 3) toxic events were documented, including 5 acute grade 3 events (e.g. brain edema) and 31 late grade ≥ 3 events (visual deterioration, cataract, radiation necrosis, brain edema, hypopituitarism and other endocrine/neurologic deficits), corresponding to a minimum crude high-grade event rate of 3% in the pooled cohort. Only one grade 4 toxicity was reported. This indicates that, while treatment-related complications are not rare overall, severe (grade ≥ 3) toxicities were uncommon.

### Dose, grade, and high-grade toxicity

Across series reporting both histology and dose, WHO Grade I meningiomas were typically treated with conventionally fractionated proton therapy of approximately 50.4–56 Gy, whereas WHO Grade II/III meningiomas more often received dose-escalated schedules of 60–70.2 Gy or hypofractionated stereotactic regimens, which were 5–6 Gy per fraction [16, 21]. In the largest mixed-grade cohorts, where WHO II/III tumors received higher doses (60–66 Gy), grade ≥ 3 late toxicities and radiation necrosis clustered predominantly in these higher-risk, higher-dose groups (up to 24 grade ≥ 3 late events reported), while Grade I–dominant series treated at 50–54 Gy reported few or no grade ≥ 3 events, typically 0–1 high-grade toxicity or radionecrosis per cohort [16, 21].

### Meta-analytic outcomes

**Fig. 2:**
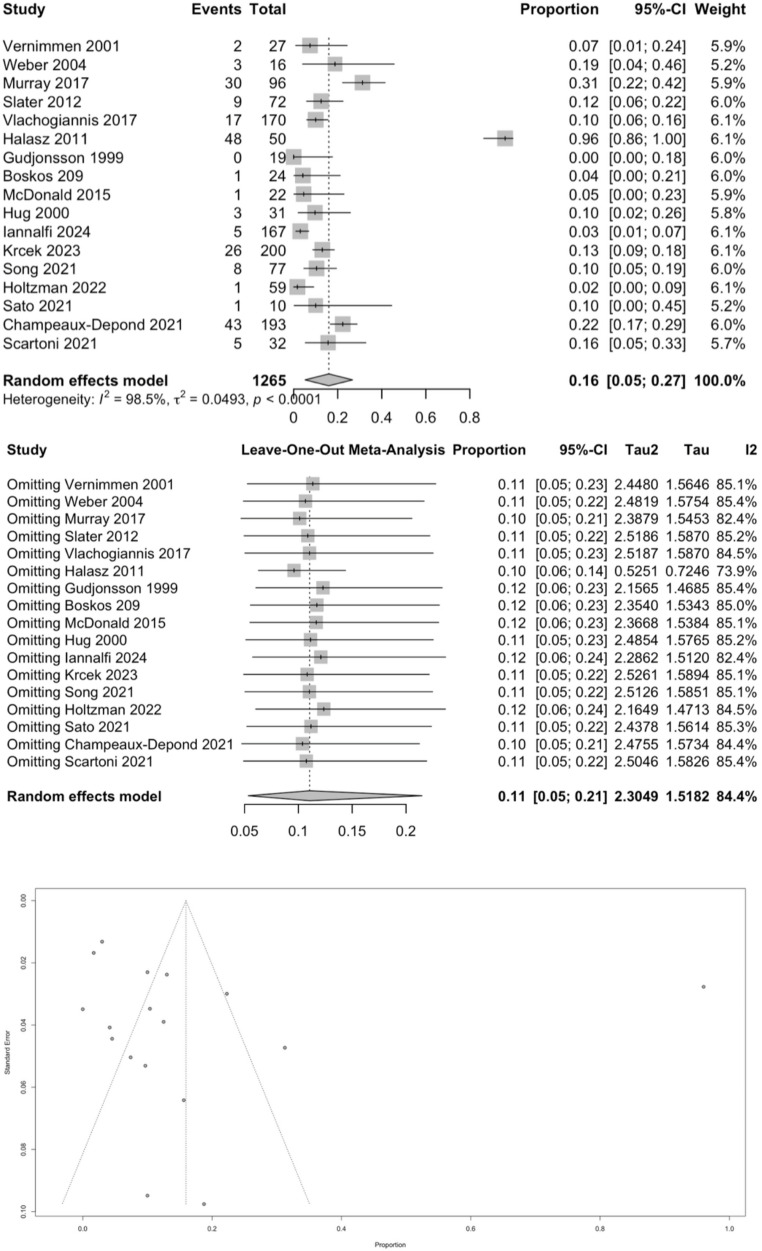
Meta-analysis of complication rates after proton therapy for intracranial meningioma

From the meta-analysis (Table 3), the pooled overall treatment-related complication rate was 16% (95% CI 0.05–0.27; *p* < 0.0001), which was statistically significant despite substantial heterogeneity (I² = 98.5%, Tau² = 0.0493) (Fig. 2). Egger’s tests showed t = −0.55 (df = 15, *p* = 0.5891) and z = −1.3783 (*p* = 0.1681), and Begg’s tests showed z = 0.33 (*p* = 0.7417) and Kendall’s tau = 0.0588 (*p* = 0.7765), all indicating no statistically significant funnel plot asymmetry. A leave-one-out sensitivity analysis revealed that the omission of Halasz 2011 substantially reduced heterogeneity to I² = 73.9% (Tau² = 0.5251) and lowered the pooled proportion to 10% (95% CI 0.06–0.14), indicating this single study was a significant outlier.

**Table 3:** Summary of meta-analytic outcomes

Outcome	*n* (patients)	Pooled estimate (95% CI)	*p*-value	I² (%)	Egger’s test	Begg’s test
Overall treatment-related complications (acute + late)	1,431	16% (5–27%)	< 0.001	98.5	z = −1.3783 (*p* = 0.1681)	Kendall’s τ = 0.0588 (*p* = 0.7765)
5-year overall survival	747	91% (88–94%)	< 0.001	49.3	z = 1.0539 (*p* = 0.2919)	Kendall’s τ = 0.1667 (*p* = 0.6122)
Radiologic local control	212	71% (50–86%)	0.002	73.1	z = 2.3837 (*p* = 0.0171)	Kendall’s τ = 1.0000 (*p* = 0.3333)

**Fig. 3:**
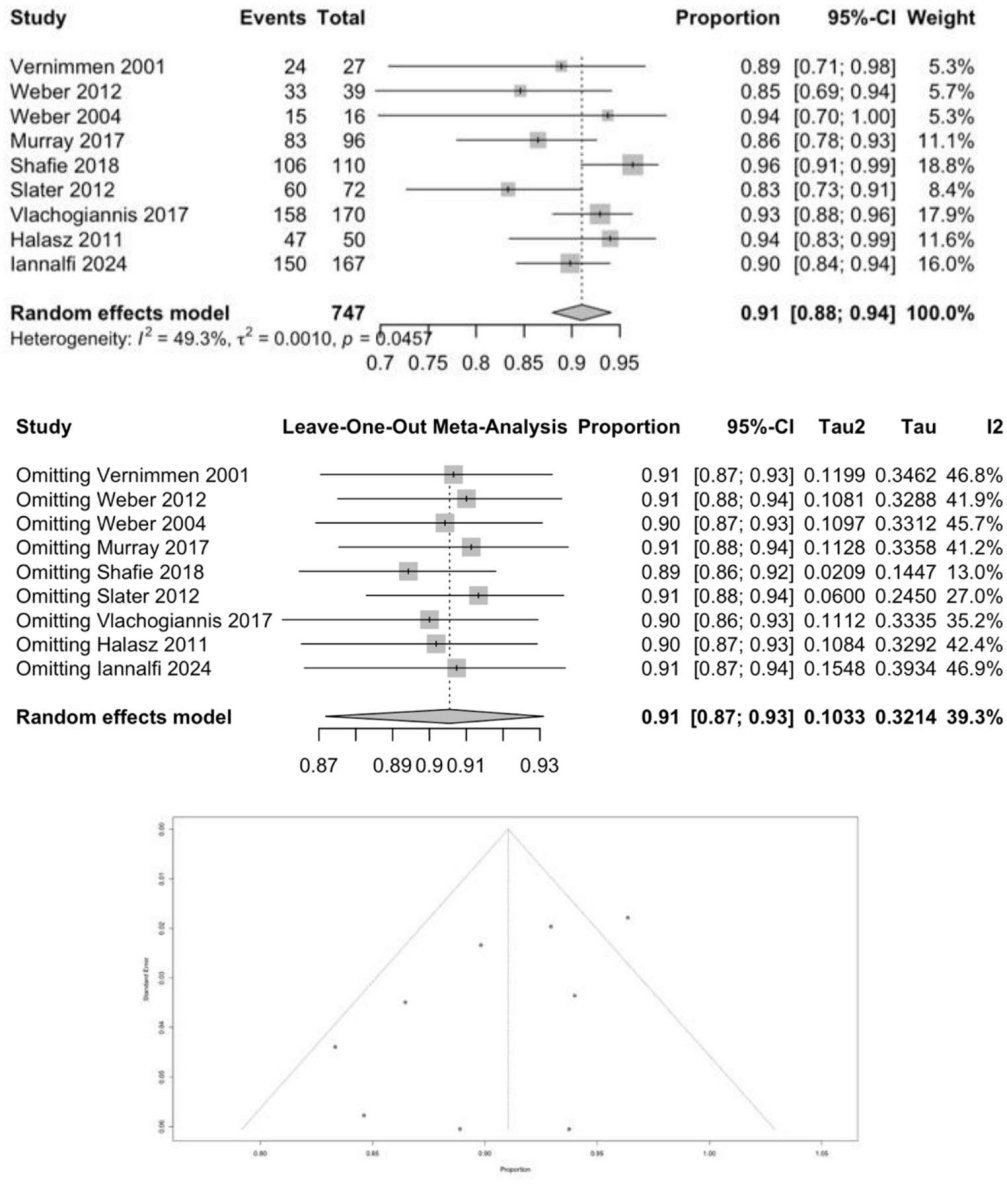
Meta-analysis of 5-year overall survival in proton-treated intracranial meningioma

Nine studies (*n* = 747) reported a statistically significant 5-year overall-survival proportion of 91% (95% CI 0.88–0.94; *p* = 0.0457; I² = 49.3%), confirming durable disease control across centers (Fig. 3). Egger’s test showed z = 1.0539 (*p* = 0.2919) and Begg’s test showed Kendall’s tau = 0.1667 (*p* = 0.6122). Leave-one-out sensitivity analysis demonstrated high stability of this pooled estimate, with overall proportions remaining stable between 0.89 and 0.91 regardless of the study omitted. However, the removal of Shafie 2018 resolved a substantial portion of the statistical heterogeneity, dropping I² to 13.0% (Tau² = 0.0209) and yielding a pooled proportion of 89% (95% CI 0.86–0.92). Long-term (≥ 10-year) survival was reported in only a small subset of studies, with heterogeneous and often non-extractable data, precluding a reliable pooled 10-year survival estimate.

**Fig. 4:**
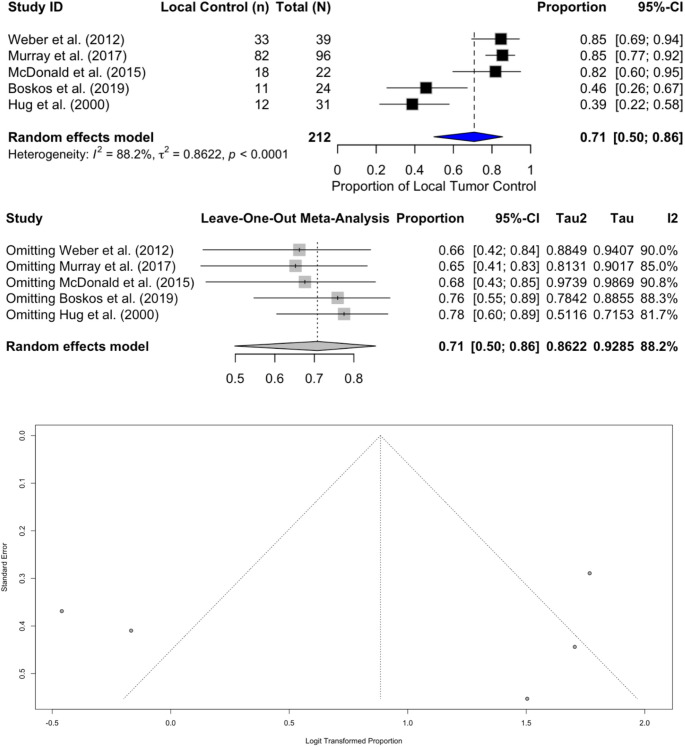
Meta-analysis of radiologic local tumor control following proton therapy for intracranial meningioma

Radiologic local control, available in five historical series (*n* = 212), averaged 71% (95% CI 0.50–0.86; *p* < 0.0001) and remained statistically significant despite notable heterogeneity (I² = 88.2%, Tau² = 0.8622) (Fig. 4). Egger’s test showed z = 2.3837 (*p* = 0.0171, intercept b = −5.3710, 95% CI −9.8073 to −0.9347), indicating statistically significant funnel plot asymmetry. Given the smaller number of included studies, influence analysis showed this estimate was highly sensitive to individual study removal. Specifically, omitting Hug et al. (2000) increased the pooled local control estimate to 78% (95% CI 0.60–0.89) and reduced heterogeneity to I² = 81.7% (Tau² = 0.5116). Similarly, omitting Boskos et al. (2019) resulted in a pooled estimate of 76% (95% CI 0.55–0.89; I² = 88.3%, Tau² = 0.7842).

### Risk of bias

**Fig. 5:**
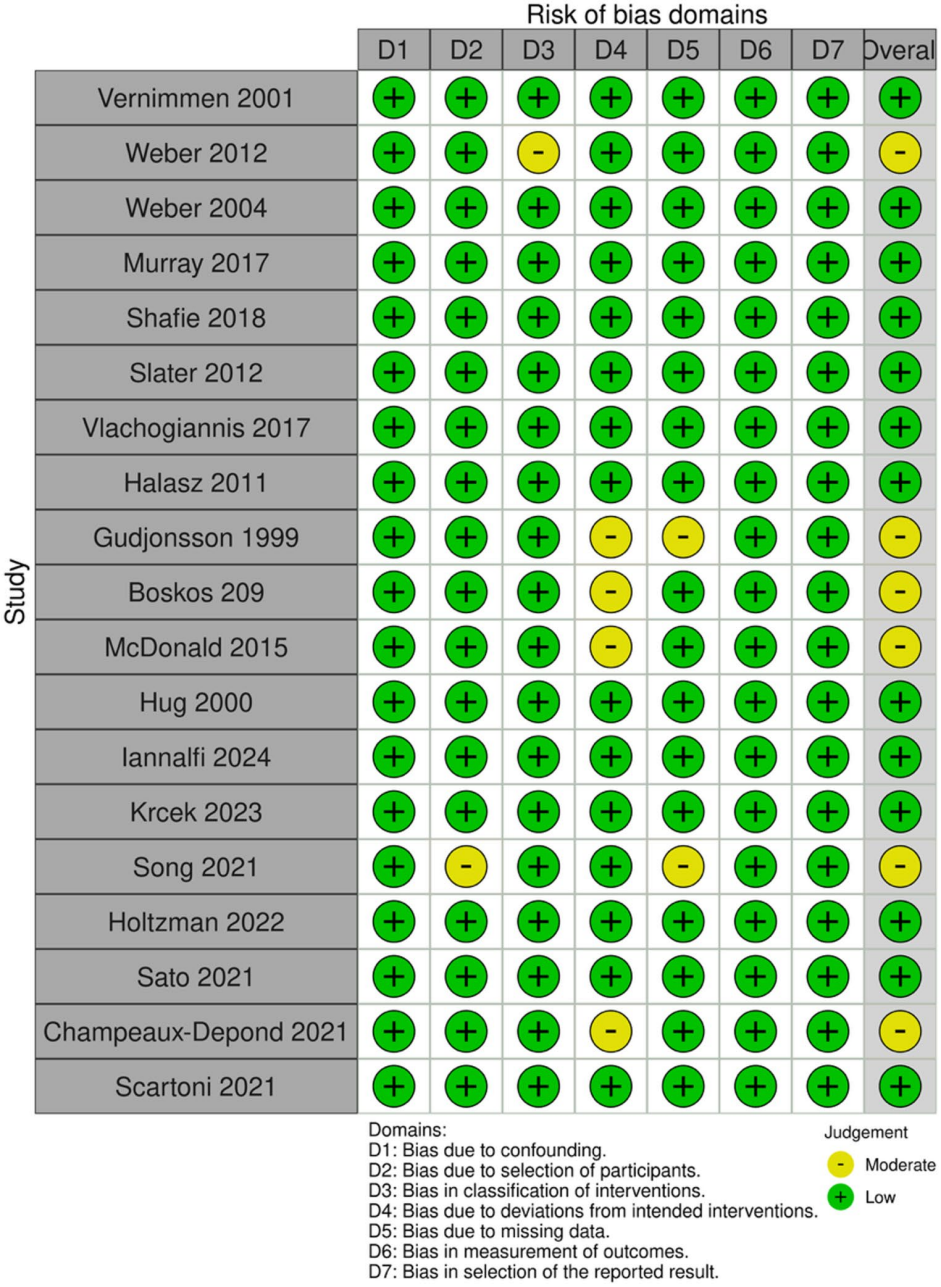
ROBINS-I risk-of-bias assessment for included studies. D1: bias due to confounding; D2: bias due to selection of participants; D3: bias in classification of interventions; D4: bias due to deviations from intended interventions; D5: bias due to missing data; D6: bias in measurement of outcomes; D7: bias in selection of the reported result. Green circles indicate low risk of bias; yellow circles indicate moderate risk of bias

The ROBINS-I appraisal demonstrated a broadly sound methodology: 13 of 19 studies were judged to have low risk of bias, whereas 6 exhibited an overall moderate risk of bias (Fig. 5). Moderate ratings clustered in D4 and D5, while every cohort retained low risk for confounding, intervention classification, outcome measurement, and selective reporting.

## Discussion

The current study found that proton therapy demonstrates high local-control rates in most series while maintaining low complication profiles. The direction and magnitude of effect were remarkably uniform across diverse clinical settings. The small-study effects test did not suggest major publication bias.

Our study shows that proton therapy for intracranial meningioma yields a pooled clinically significant complication rate of 16% (95% CI 5–27; *p* < 0.001), with adverse events predominantly graded ≤ 2 toxicity. Supporting evidence from large photon-based IMRT series frequently cites late grade ≥ 3 toxicity rates of 25–30%, whereas single-isocentre stereotactic radiosurgery sometimes reports single-digit morbidity. These data suggest that proton-related complication rates fall between these two radiation techniques [26]. Conversely, a smaller Japanese passive-scatter experience described only 9% grade ≥ 3 toxicity events, highlighting how delivery technique and case mix modulate toxicity and partially explain the marked heterogeneity (I² = 99%) [27]. The Bragg-peak’s steep distal fall-off and negligible exit dose reduce integral cranial exposure. However, residual uncertainties in range, spot size, and neutron contamination, particularly in older passive-scatter units, can still precipitate optic neuropathy or radionecrosis if planning margins are inadequate [28, 29]. Contemporary pencil-beam scanning with robust optimization and smaller lateral penumbra is therefore expected to drive complication rates further downward by sharpening dose gradients and dampening high-LET spill into normal tissue [29].

Five-year overall survival was 91% (95% CI 88–94; *p* < 0.001) of the reported cohort. Across proton-beam series, 5-year overall survival ranges 87–100% overall, 93–100% for WHO I, 77–89% for WHO II, and 44–52% for WHO III, consistent with our pooled OS [30]. When compared with maximum safe surgical resection, which achieves approximately 92–94% 5-year OS for resectable low-grade tumors, and stereotactic radiosurgery, with reported 5-year OS around 88–92%, proton therapy demonstrates broadly comparable survival. This is particularly true for WHO I and II lesions and is notable because proton therapy offers a non-invasive option in surgically challenging or medically inoperable cases [31]. Radiobiologically, protons can deliver a curative dose with high conformity while sparing hippocampal and cerebrovascular territories whose late damage contributes to neurocognitive decline and vascular mortality. This organ sparing theoretically augments both the duration and quality of survival beyond the current five-year observation window [32]. The lower integral dose mitigates secondary-malignancy risk, a benefit not yet detectable in the included datasets but expected to manifest over decades of follow-up [32].

Radiologic local-tumor control was reported in 71% (95% CI 50–86; *p* = 0.002). This pooled estimate reflects radiologic local control defined as stability or regression on serial CT/MRI and is derived from three older series enriched for higher-risk or higher-grade meningiomas with long follow-up. Heterogeneity in imaging protocols and progression criteria across these studies likely contributed to both the comparatively low control estimate and the observed statistical heterogeneity. In comparison, contemporary series using advanced photon and carbon-ion techniques generally report 5-year local control rates in the range of 70–90%. This suggests that our pooled proton LC estimate of 52% mainly reflects older, higher-risk cohorts and heterogeneous imaging criteria in the historical studies included in this analysis [33]. Conversely, the Mayo passive-scatter experience noted progression in 40% of atypical meningiomas despite dose escalation, indicating that RBE uncertainty and aggressive biology can erode proton efficacy in selected subgroups [34]. A distal-range uncertainty of 2–3% of beam energy can translate into millimetric under-coverage near skull-base critical structures. When compounded by tumor shrinkage or cerebrospinal fluid shifts, residual clonogens may persist and repopulate long after treatment [34].

### Pencil beam proton therapy

In the proton literature using pencil beam or spot-scanning delivery, long-term local control is generally high, with pencil beam scanning cohorts in our dataset clustering around 0.85. In contrast, when all proton-era techniques and mixed-risk populations are pooled, our gold-standard meta-analysis yields an overall local tumor control proportion of 0.71 (95% CI 0.50–0.86) with substantial heterogeneity (I²=88.2%). For photon “classic” benchmarks, a recent SRS single-arm meta-analysis in large-volume meningiomas reported pooled local control 91% (95% CI 86–94) and pooled 5-year PFS 92% (95% CI 86–96) [35]. For fractionated photon therapy, an early IMRT series reported 93% 5-year local control, and a later skull-base high-precision RT cohort reported 92% local control at 5 years [36]. Many candidates for proton therapy are skull-base or residual or recurrent tumors where further resection risks cranial nerve or vascular morbidity, while from an engineering standpoint pencil beam scanning enables intensity-modulated proton delivery with steep distal fall-off and reduced integral dose to adjacent organs-at-risk.

### Study limitations

The methodological limitations of this review include an evidence base dominated by single-institution, retrospective cohorts collected between 1999 and 2024. This extended timespan encompasses significant evolution in proton-beam hardware, transitioning from passive-scatter units to pencil-beam-scanning gantries with smaller lateral penumbra. The clinical heterogeneity accompanying this technical drift blurs the true signal of modern practice, particularly for convexity or parasagittal meningiomas, which may profit less from Bragg-peak conformality compared to the skull-base lesions that constitute roughly four-fifths of our pooled sample. Furthermore, the extent of surgical cytoreduction varied widely across series, yet few studies stratified outcomes by extent of resection. Lastly, long-term (≥ 10-year) survival was reported infrequently and in heterogeneous, often non-extractable formats, preventing robust meta-analysis beyond 5-year outcomes and limiting our ability to draw firm conclusions regarding very late disease control and toxicity.

Regarding reporting limitations, imaging follow-up protocols ranged from planar CT to high-resolution volumetric MRI, producing discordant definitions of “local control” and undercutting cross-study comparability [9]. Key late outcomes were reported inconsistently or omitted entirely, limiting insight into the full late-effect spectrum that theoretically differentiates protons from photons. Crucially, the meta-analytic estimates for complications (I^2^ = 98.5%) and local control (I^2^ = 88.2%) are encumbered by extreme between-study heterogeneity. This inflates random-effects confidence intervals and demands a cautious interpretation of any single pooled proportion. With only a few studies informing the local-control model, there was insufficient power for meta-regression to explore dose, fractionation, or histology as moderators.

### Future directions

Future work should prioritize multicenter, prospective cohorts with standardized reporting of clinical, dosimetric, and surgical variables. Harmonized definitions of local control, prospectively collected late toxicity graded with contemporary systems (e.g., CTCAE), and routine reporting of grade ≥ 3 events would substantially reduce measurement bias and strengthen future meta-analytic inferences. Larger, more granular datasets would also enable adequately powered meta-regression to test candidate moderators such as total dose, WHO grade, treatment year (technology era), and fractionation type. In parallel, collaborative registries and, where feasible, randomized or pragmatic comparative trials against modern photon IMRT and stereotactic radiosurgery could validate the survival and toxicity advantages suggested by this review and inform cost-effectiveness across different healthcare settings.

## Conclusion

Across 1,431 patients, the pooled data suggests a 5-year overall survival of approximately 91% for Grade I and 70–85% for Grade II/III meningiomas, alongside an overall complication rate of 16%. While these figures indicate that proton therapy is associated with favorable local control and overall survival with a relatively low incidence of severe late toxicities, the high degree of statistical and clinical heterogeneity across the included retrospective datasets necessitates cautious interpretation. Although higher radiation doses appear beneficial for controlling Grade II/III tumors, this potential benefit must be carefully weighed against the risk of neurotoxicity on a case-by-case basis. These findings highlight the potential utility of integrating proton therapy into multidisciplinary treatment planning, particularly for complex cases where tissue sparing is paramount. Ultimately, larger multicenter prospective cohorts, collaborative registries, and pragmatic randomized trials are essential to validate these findings, refine dose protocols, evaluate long-term outcomes, and establish cost-effectiveness across evolving proton technologies and diverse healthcare settings.

## Data Availability

The datasets generated during and/or analysed during the current study are available from the corresponding author on reasonable request.
