# Correction to: Effectiveness and safety of proton therapy in intracranial meningioma treatment: a systematic review and meta-analysis

**DOI:** 10.1007/s10143-026-04339-1

**Published:** 2026-05-26

**Authors:** Jeremiah Hillkiah Wijaya, Abdelrahman Ramadan Elashry, Sarmad Javaid, Mohga Yasser, Bismon Jibu, Bharath Narayanan, Daniela A. Perez-Chadid, Aafreen Azmi, Juan Pablo Avila-Madrigal, Elizabeth E. Ginalis, Anil Nanda

**Affiliations:** 1 School of Public Health and Preventive Medicine, Monash University, Melbourne, VIC Australia; 2 College of Medicine, University of Sharjah, Sharjah, UAE; 3 Sharif Medical and Dental College, Lahore, Pakistan; 4 Far Eastern Medical College, Khabarovsk, Russia; 5 Department of Neurosurgery, Rutgers New Jersey Medical School, Rutgers University, Newark, NJ USA; 6 Department of Neurosurgery, Robert Wood Johnson University Hospital, Robert Wood Johnson Medical School, Rutgers University, New Brunswick, NJ USA; 7 Anatomy Department, Universidad de los Andes School of Medicine, Bogota, Colombia

**Correction to: Neurosurgical Review**

10.1007/s10143-026-04319-5

The authors regret that the name of Dr. Mohga Yasser was incorrectly listed as Mohga Khaled in the original published article. His affiliation also needs to be corrected to ^2^College of Medicine, University of Sharjah, Sharjah, UAE.

The original article online has been corrected.

